# Imaging of temperature dependent hemodynamics in the rat sciatic nerve by functional photoacoustic microscopy

**DOI:** 10.1186/1475-925X-12-120

**Published:** 2013-11-18

**Authors:** Lun-De Liao, Josue Orellana, Yu-Hang Liu, Yan-Ren Lin, Ashwati Vipin, Nitish V Thakor, Kaiquan Shen, Einar Wilder-Smith

**Affiliations:** 1 Singapore Institute for Neurotechnology (SINAPSE), National University of Singapore, 28 Medical Drive, #05-COR, Singapore 117456, Singapore; 2 Department of Biomedical Engineering, Johns Hopkins University, Traylor 701/720 Rutland Ave, Baltimore 21205, USA; 3 Department of Emergency Medicine, Changhua Christian Hospital, Changhua, Taiwan; 4 School of Medicine, Chung Shan Medical University, Taichung, Taiwan; 5 Department of Medicine, Yong Loo Lin School of Medicine, National University of Singapore, Singapore 117456, Singapore

**Keywords:** Peripheral nerve imaging, Functional photoacoustic microscopy (fPAM), Temperature stimulation, Total hemoglobin concentration (HbT), Hemoglobin oxygen saturation (SO_2_)

## Abstract

**Background:**

Vascular hemodynamics is central to the regulation of neuro-metabolism and plays important roles in peripheral nerves diseases and their prevention. However, at present there are only a few techniques capable of directly measuring peripheral nerve vascular hemodynamics.

**Method:**

Here, we investigate the use of dark-field functional photoacoustic microscopy (fPAM) for intrinsic visualizing of the relative hemodynamics of the rat sciatic nerve in response to localized temperature modulation (i.e., cooling and rewarming).

**Results and conclusion:**

Our main results show that the relative functional total hemoglobin concentration (HbT) is more significantly correlated with localized temperature changes than the hemoglobin oxygen saturation (SO_2_) changes in the sciatic nerve. Our study also indicates that the relative HbT changes are better markers of neuronal activation than SO_2_ during nerve temperature changes. Our results show that fPAM is a promising candidate for *in vivo* imaging of peripheral nerve hemodynamics without the use of contrast agents. Additionally, this technique may shed light on the neuroprotective effect of hypothermia on peripheral nerves by visualizing their intrinsic hemodynamics.

## Introduction

Visualizing hemodynamic changes through total hemoglobin concentration (HbT) and hemoglobin oxygen saturation (SO_2_) can lead to high impact *in vivo* research and clinical applications in the treatment and prevention of peripheral nerve diseases [1-5]. These include the study of thermoregulatory sensory pathways [6,7], peripheral nerve injury [3,8,9] and disease models such as neuropathy [1,10]. Medical ultrasound imaging (MUI) and functional magnetic resonance imaging (fMRI) are the two most prominent techniques for imaging the structure and function of peripheral nerve, respectively [3]. MUI provides real-time blood flow and morphological information of the nerve. Liu et al. applied high-frequency MUI to evaluate morphological changes in human diabetic neuropathy by visualizing the relative change in the fascicular size of sural nerves [3]. Moreover, Jan et al. used laser Doppler to investigate the skin blood flow response to locally applied mechanical and thermal stresses for the study of diabetic neuropathy [11]. However, label-free bio-tissue imaging of physiological parameters (i.e., SO_2_) using MUI or laser Doppler is still challenging [3,11]. In contrast, fMRI maps hemodynamics by measuring surrogate changes in blood flow and oxygenation, which accompany changes in neural activity. Probing the hemodynamics down to the level of a single nerve group and its nurturing blood vessels without the use of contrast agents is difficult to achieve for most of other imaging techniques, including fMRI [12].

Recently, optical imaging has been increasingly used to study hemodynamics *in vivo*[13,14]; the optical spectroscopy method can probe oxy- and deoxy-hemoglobin (i.e., HbO_2_ and Hb) through its distinct optical absorption characteristics [13,15-17]. But, due to light’s limited penetration depth [14,18], optical imaging techniques can only probe information from the surface level of target blood vessels [19-21] and provide no structural information for the deeper tissue [14,22]. Though current MUI, laser Doppler, fMRI and optical imaging techniques can register vascular responses by using their intrinsic contrast, the capability of label free study of *in vivo* hemodynamics at the peripheral nerve level is still beyond their conventional use [18].

Photoacoustic (PA) is a novel optical absorption-based hybrid imaging methodology that combines the merits of ultrasound and optical imaging techniques and allows for the monitoring of intrinsic hemodynamic changes with a deep penetration reach [23,24] and without the use of contrast agents. PA imaging technique has three major implementations: dark-field PA microscopy (PAM), PA computed tomography (PAT) and PA endoscopy (PAE). Whereas PAM and PAE usually aim to image millimeters deep at micrometer-scale resolution, PAT can be implemented for deeper imaging ability but only with few hundred micrometer-scale resolution. A reflection-mode functional PAM (fPAM) has been applied to subcutaneous vasculature [24], tumor detection of breast [25] and brain [26], oxygenation monitoring in blood vessels and neurovascular imaging [23,27,28] in small animal models.

Here, we report dark-field fPAM as a reliable imaging technique for the label-free study of *in vivo* relative hemodynamics in the rat sciatic nerve during temperature modulation via cooling and rewarming. The fundamental thermoregulatory implications of localized nerve temperature changes have not been fully elucidated [8]. For the first time, we demonstrate this methodology for *in vivo* visualization of the relative hemodynamics in the rat sciatic nerve. We studied and characterized the temperature dependent vascular dynamics of the rat sciatic nerve under targeted cooling and rewarming conditions. This methodology has significant potential for enhancing our understanding of nerve hemodynamics as well as providing novel insights for evaluating the vascular environment under various stimulation conditions, especially involving peripheral nerve diseases in small animal models [29].

## Materials and methods

### Dark-field confocal functional photoacoustic microscopy system

**Figure 1:**
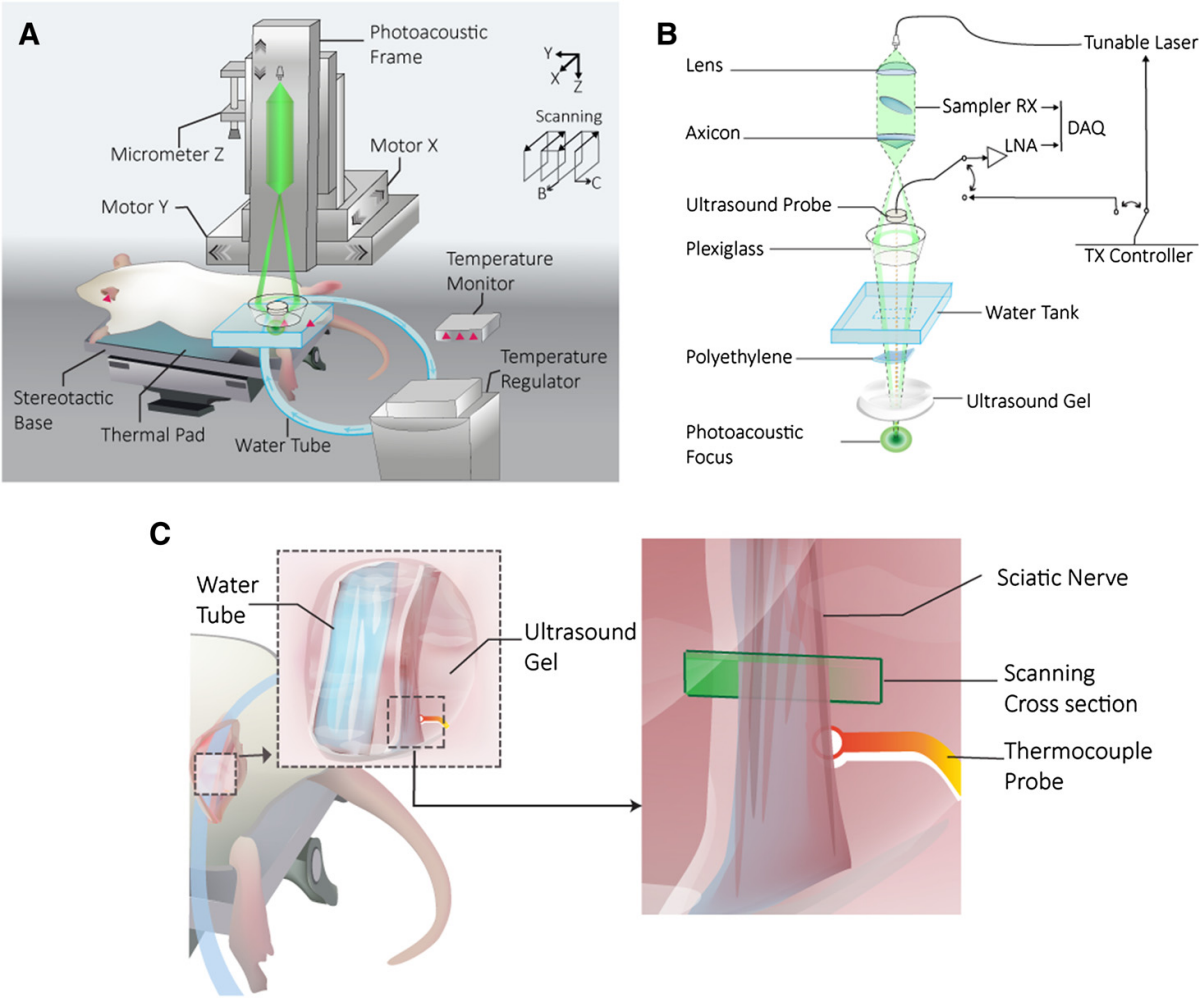
**PA experiment setup of this cooling study. (A)** Experimental dark-field fPAM system integrated with a thermoregulation setup. Commercially available ultrasound gel was applied on the rat sciatic nerve for acoustic and thermal coupling; the rat subjects were placed between the water container and a custom-made stereotaxic apparatus for imaging. **(B)** The laser was pulsed with frequency of 10 Hz and coupled to an optical fiber into the strong focusing dark-field PA path to illuminate the target cross-section at the nerve. PA waves were detected by a 50-MHz transducer and then through the A/D card to the computer for further data analysis. **(C)** Localized temperature modulation was achieved via immediate heat transfer between the sciatic nerve and the perfused thermoregulatory water tube. A fixed scanning cross-section was selected during all experiments. The nerve thermocouple couple probe was placed directly below the sciatic trifurcation. We also applied sutures as needed to reinforce the stability of the tube and thermocouple probe.

Our 50-MHz dark-field confocal fPAM system for imaging functional hemodynamics in the sciatic nerve is shown in Figure 1 consisting of laser pulse generation and delivery (Figure 1A), PA signal reception, and image reconstruction and display (Figure 1B). Laser pulses, 4 ns wide, were generated at a frequency of 10 Hz by using an optical parametric oscillator (Surlite OPO Plus, Continuum, USA). The laser was pumped by a frequency-tripled Nd:YAG Q-switched laser (Surlite II-10, Continuum, USA). Two visible wavelengths of the laser pulses, 560 and 570 nm (λ_560_ and λ_570_), were employed for PA wave excitation [27]. At the selected wavelengths, blood is a dominant optical absorber, producing strong optical absorption and thus guaranteeing that the detected PA signals mainly come from blood [23,27]. The acquired PA signal at λ_560_ is sensitive to relative changes in SO_2_, while relative HbT changes are the most prominent at λ_570_[23]. The 50-MHz ultrasonic transducer used in the current fPAM system was custom-made by the Acoustic Sensor Co., Ltd at Taiwan. It has a −6 dB fractional bandwidth of 57.5%, a focal length of 9 mm and a 6 mm active element, offering an axial resolution of 32 μm and a lateral resolution of 61 μm.

Laser energy was delivered using a 1-mm multimodal fiber (Thorlabs, U.S.A). The fiber tip was coaxially aligned with a convex lens, an axicon, a plexiglass mirror, and an ultrasonic transducer on an optical bench, forming dark-field illumination that was confocal with the focal point of the ultrasonic transducer. The incident energy density on the sample surface was well within American National Standards institute (ANSI) safety limits. The transducer was immersed in an acrylic water tank during the imaging process, and the hole at the bottom of the tank was sealed with a piece of 15-μm thick polyethylene film. A thin layer of ultrasonic gel was applied as a PA and thermal conductive medium, which was then attached to the thin polyethylene film to ensure reliable coupling of the PA waves with the water tank. The PA signals received by the ultrasonic transducer were pre-amplified by a low-noise amplifier (noise figure 1.2 dB, gain 55 dB, AU-3A-0110, USA), cascaded to an ultrasonic receiver (5073 PR, Olympus, USA) and then digitized and sampled by a computer-based 14-bit analog to digital (A/D) card (CompuScope 14220, GaGe, USA) at a 200-MHz sampling rate for data storage.

Fluctuations in the laser energy were monitored with a photodiode (DET36A/M, Thorlabs, USA). The recorded photodiode signals were measured prior to the experiment to compensate for PA signal variations caused by laser-energy instability. The achievable penetration depth of the current fPAM system was 3 mm with approximately 18-dB SNR, where SNR is defined as the ratio of the signal peak value to the root-mean-square value of the noise. Three scan types can be provided by this system (Figure 1A): A-line (i.e., one-dimensional images where the axis represents the imaging depth), B-scan (i.e., two-dimensional images where one axis is the lateral scanning distance and the other is the imaging depth), and C-scan (i.e., projection images from the three-dimensional images) [24]. The amplitude of the envelope-detected PA signals was used in the subsequent functional imaging analysis [23].

### Experimental animals

Ten adult female Wistar rats (NUS-CARE, Singapore) weighing 280 ± 20 grams were used and housed at a constant temperature and humidity with free access to food and water. The Institutional Animal Care and Use Committee (IACUC) at the National University of Singapore approved all the experimental procedures.

Rats remained anesthetized with isoflurane 2-3% in 100% O_2_ and were mounted on a dorsal position over a custom-made acrylic stereotaxic holder. Next, the left hind limb was shaved and disinfected prior to making a 40 mm longitudinal incision at knee level. The biceps femoris was detached and folded towards the posterior. Also the caudofemoralis was transected in order to completely expose the sciatic nerve [30].

A thermoregulatory device was customized to provide localized temperature modulation to the exposed rat sciatic nerve. The apparatus consisted of a flexible tube (Bev-A-Line IV, out diameter at 4.8 mm, inner diameter at 3.2 mm, thermoplastic processes, NJ, USA), which was inserted through 5 mm sub-muscular incisions and placed in parallel at about 5 mm to the left of the in situ sciatic nerve as shown in Figure 1C.

### Thermoregulation

Temperature controlled water was continually circulated through the tube for immediate cooling and subsequent rewarming of the sciatic nerve using Blanketrol II system (Cincinnati Sub-zero, OH, USA), as shown in Figure 1A and 1B. A thermal blanket was placed on the ventral surface of the animal in order to maintain core body temperature at normothermia (37 ± 0.5°C). Thermocouple probes were used to monitor tympanic, rectal and sciatic nerve temperatures (prior to trifurcation) (as indicated in Figure 1A and 1C). The temperature data were recorded at 2 Hz using Thermes USB acquisition system and proprietary software (Physitemp, NJ, USA).

**Figure 2:**
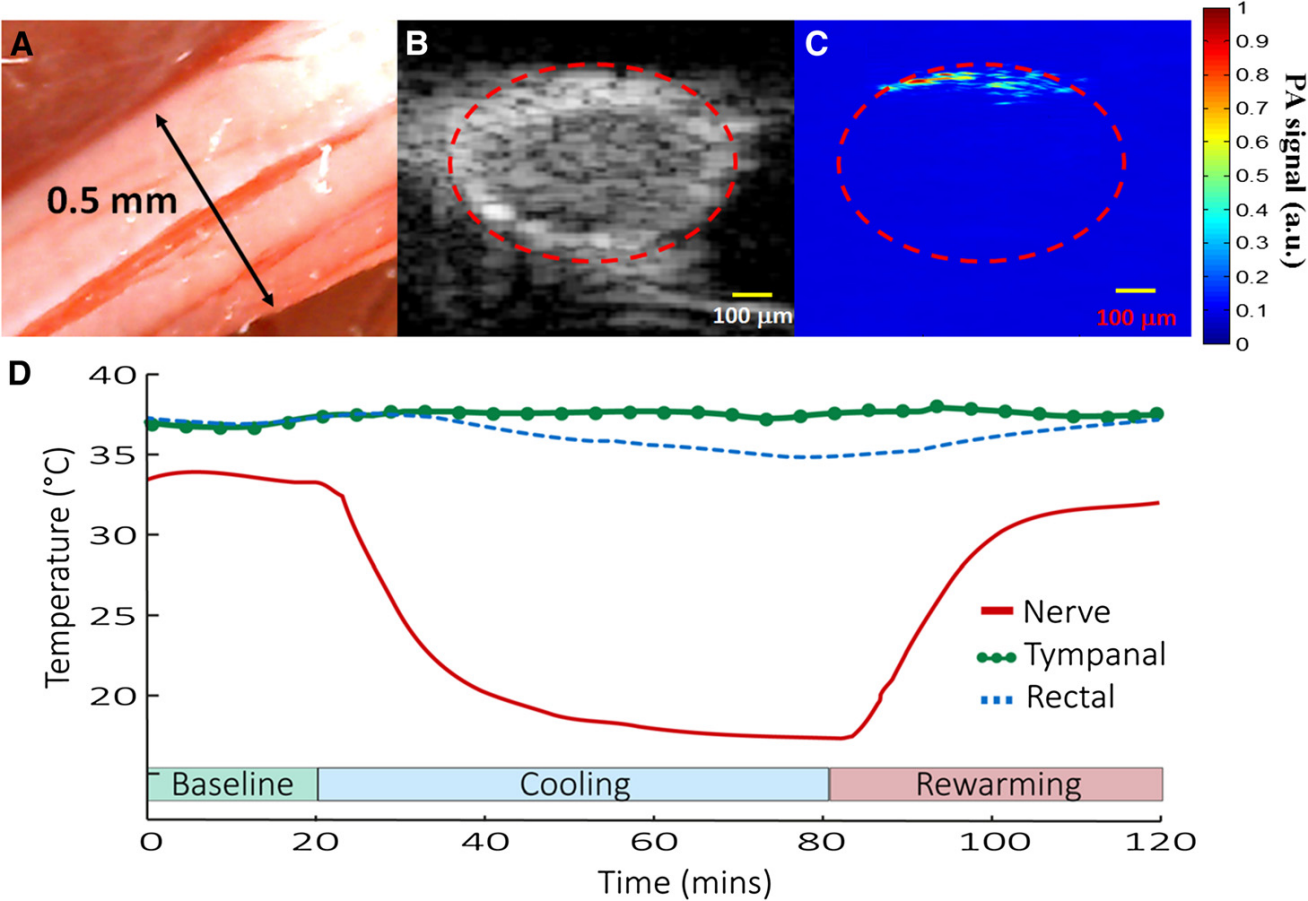
**Cooling protocol for the sciatic nerve. (A)** Photograph of the sciatic nerve (about 0.5 mm) showing several blood vessels from the epineurial vascular plexus. **(B)** Ultrasound and **(C)** PA cross-sectional B-scan images of the sciatic nerve. The yellow scale bars are equivalent to 100 μm. The ROI with the PA signal changes in scanned sciatic nerve image section was identified by the ultrasound image, as indicated by the red dashed line in **(B)**. **(D)** Localized nerve thermoregulation protocol, including the baseline, cooling and rewarming. Temperature changes in the sciatic nerve, tympanic and rectal areas were monitored.

The temperature modulation protocol consisted of three stages: baseline, cooling and rewarming for all experimental animals (Figure 2D). Baseline stage was recorded while the sciatic nerve temperature remained at 33 ± 2°C. The cooling stage began 20 minutes after the onset of baseline recording. At this point, the sciatic nerve temperature was monotonically decreased by at least 10°C at an approximate rate of −0.5°C/min. The cooling period ended after a plateau was maintained for 40 minutes. Subsequently, the sciatic nerve temperature was reverted to its baseline target during the rewarming stage, with a monotonic increase of about 0.5°C/min. The experiment concluded after 20 minutes of a maintained rewarmed state.

### Data analysis of the functional changes in HbT and SO_2_

Two optimized wavelengths (i.e., λ_560_ and λ_570_) were employed for monitoring the functional HbT and SO_2_ changes with a high SNR and sensitivity [23]. The optical absorption of blood at λ_560_ is sensitive to SO_2_ levels, while the blood absorption at λ_570_ results from the isobestic point of molar extinction spectra for oxy- and deoxy-hemoglobin [18,31]. Because the λ_570_ PA signal at a given pixel is proportional to the HbT within its resolution cell centered at that pixel, the mean functional HbT changes (*R*_HbT_(*t*)) in the selected sciatic nerve region can be assessed as follows:

(1) $$R_{\mathrm{HbT}}\left(t\right)=\frac{\sum_{\left(x,z\right)\in \mathrm{ROI}\;\mathrm{pixel}}\left(I_{\left(570\right)}\left(x,z,t\right)\right)/A\left(I_{\left(570\right)}\left(t_{0}\right)\right)}{\sum_{\left(x,z\right)\in \mathrm{ROI}\;\mathrm{pixel}}\left(I_{\left(570\right)}\left(x,z,t_{0}\right)\right)/A\left(I_{\left(570\right)}\left(t_{0}\right)\right)}\times 100\%,$$

where (x, z) is the pixel position; *I*_(570)_(x, z, *t*) is the PA image at λ_570_ acquired at time *t* and *I*_(570)_ (x, z, *t*_0_) is the baseline PA signal at λ_570_ acquired immediately before the onset of cooling (i.e. at the baseline *t*_0_); *A*(*I*_(570)_(*t*_0_)) represents the total pixel count of regions of interest (ROI) at the baseline *t*_0_[18]. Here, the ROI pixel was defined as the pixel that possessed a PA signal that was at least three times greater than the background signal [23,32].

Functional images of SO_2_ changes (*I*_*F*(560)_(*t*)) at a given time point, *t*, at each stage were assessed according to the following equation:

(2) $$I_{F\left(560\right)}\left(t\right)=\frac{I_{\left(560\right)}\left(t\right)}{I_{\left(570\right)}\left(t\right)},$$

where *I*_*(560)*_(*t*), i.e., PA image acquired at λ_560_, was normalized to *I*_*(570)*_(*t*) on a pixel-by-pixel basis [18]. The mean functional SO_2_ changes $\left(R_{SO_{2}}\left(t\right)\right)$ in a single ROI region during the stimulation period were probed as follows:

(3) $$R_{\mathrm{SO}}\left(t\right)=\frac{\sum_{\left(x,z\right)\in \mathrm{ROI}\;\mathrm{pixel}}\left(I_{F\left(560\right)}\left(x,z,t\right)\right)/A\left(I_{\left(570\right)}\left(t_{0}\right)\right)}{\sum_{\left(x,z\right)\in \mathrm{ROI}\;\mathrm{pixel}}\left(I_{F\left(570\right)}\left(x,z,t_{0}\right)\right)/A\left(I_{\left(570\right)}\left(t_{0}\right)\right)}\times 100\%.$$

That is, an independent probing of the changes in HbT and SO_2_ could be achieved where *I*_*(570)*_ was used as a marker for HbT, and *I*_*F(560)*_ was used as a marker for SO_2_[18,23,33].

This experiment was designed to quantitatively compare the differences in relative PA signal changes from temperature modulation in the vasculature of the rat sciatic nerve. In the current fPAM setting, the data acquisition time for each PA B-scan image with 31 scanned lines (2 mm width) is about 28 seconds. Hence, it takes about 56 seconds for one functional image of *I*_*F*(560)_. To identify vascular changes in response to temperature modulation, functional ultrasound and PA images were registered at a fixed cross-sectional area. Images acquired from the ultrasound scanning of the sciatic nerve were used as a reference to identify morphological characteristics and the PA region of interest (ROI) as indicated by the red dashed line in Figure 2B and 2C.

Statistical significance was assessed using a paired *t*-test with significance defined as *p*-value of < 0.05 for the side-to-side differences in PA signals (*I*_*R(570)*_ and *I*_*F*(560)_) of the studied areas. The significance of changes observed in fPAM signals (*I*_*F*(560)_) at the respective ROI in response to temperature modulation was compared using the Wilcoxon matched-pairs signed-rank test (two-tailed, *p* < 0.05, *n* = 10) [23,27,33]. All statistical analyses were performed using SPSS (version 10.0, SPSS®, USA).

## Results

### PA imaging of the rat sciatic nerve vasculature

A photograph of the surface of rat sciatic nerve is shown in Figure 2A. Many distinct blood vessels varying in size can be seen at the epineurium of the sciatic nerve. The B-scan ultrasound and PA images of the rat sciatic nerve are shown in Figure 2B and 2C, respectively. Increased relative HbT and SO_2_ values peak in the same areas that the blood vessel are seen, suggesting that these regions represent blood vessels as visualized by fPAM (Figure 2C).

### Localized thermoregulation response

**Figure 3:**
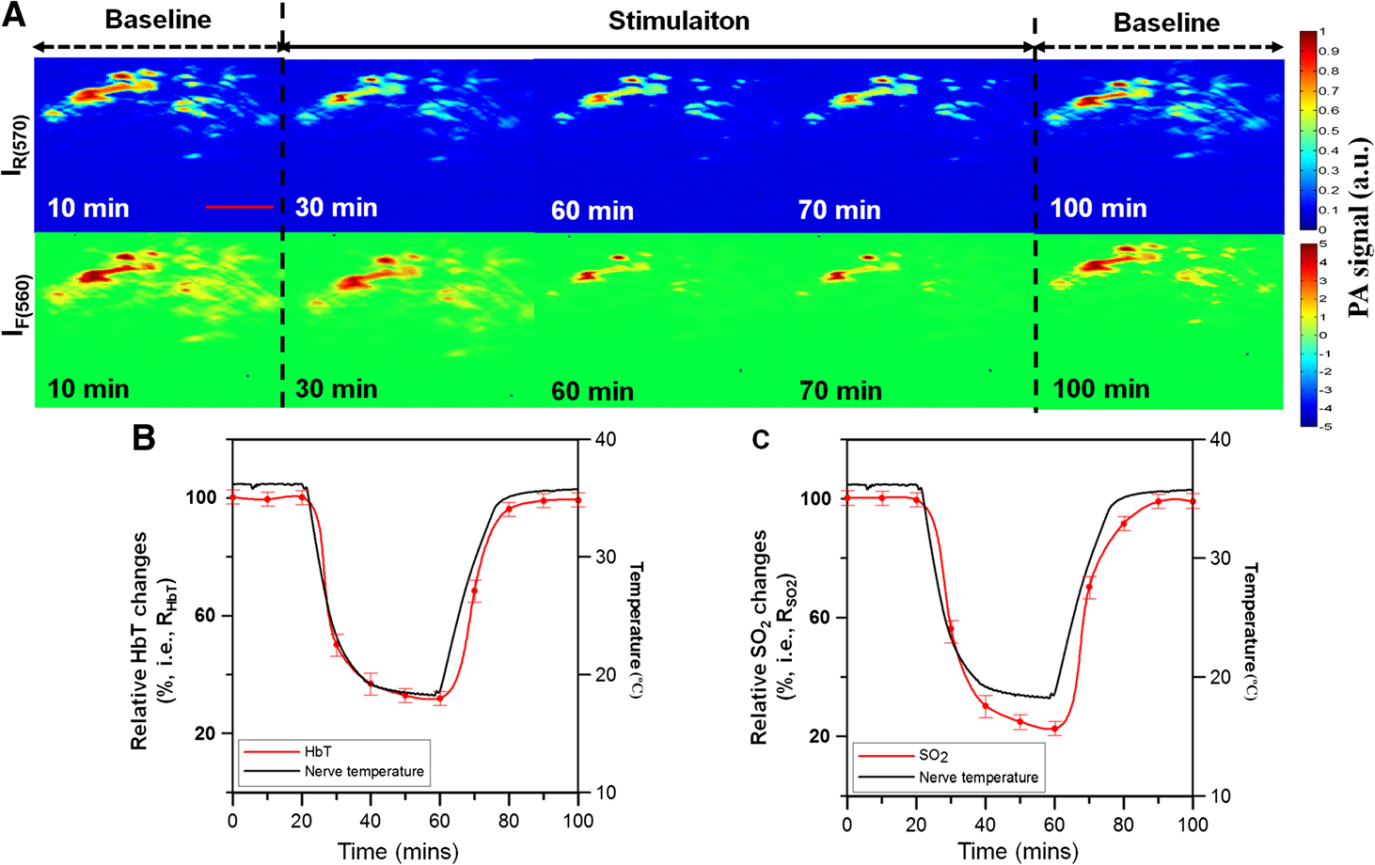
**PA imaging results. (A)***In vivo* relative *I*_*(570)*_ (i.e., HbT; upper panel) and *I*_*F*(560)_ (i.e., SO_2_; lower panel) PA B-scan images of selected position at different times of temperature modulation protocol**.** Note that the *I*_*(570)*_ and *I*_*F*(560)_ are specifically sensitive to relative HbT and SO_2_ changes, respectively. The red scale bar is equivalent to 100 μm and applies to all images in panel **A**. Mean functional **(B)** HbT (i.e., *R*_*HbT*_) and **(C)** SO_2_ (i.e., $R_{SO_{2}}$) changes resulting from *in vivo* temperature modulation of the rat sciatic nerve as a function of time. The error bars indicate standard deviation (*n* = 10).

A selected cross-section region of the sciatic nerve was examined for relative side-to-side PA signal differences in response to localized temperature modulation. The thermoregulation protocol was designed to quantitatively compare the relative HbT and SO_2_ changes in vascular structures of the sciatic nerve between three localized temperature modulation stages: baseline, cooling and rewarming (Figure 2D). Figure 3A and Additional file 1: Movie S1 show the response for both relative HbT changes as function of time under the thermoregulation protocol.

### Functional temperature dependent hemodynamics in sciatic nerve

*In vivo* functional hemodynamics of the sciatic nerve in response to thermoregulation at difference stages are shown in Figure 3A. The ultrasound and PA images are shown in Figure 2B and 2C, respectively, and their ROIs were used for the statistical analysis. Both relative HbT and SO_2_ changes demonstrate significant correlations with localized thermoregulation during cooling and rewarming stages, as shown in Figure 3B and 3C, respectively (*p* < 0.05; paired *t*-test). Quantitative analysis shows that during cooling, relative HbT (*R*_*HbT*_) was reduced by −70.8% with respect to its baseline, and correlated with temperature by r=0.94. Similarly, relative SO_2_$\left(R_{SO_{2}}\right)$ was lowered by −73.3% from its baseline value, and correlated with temperature by r=0.88. The applied thermoregulation protocol did not significantly affect *R*_*HbT*_ and $R_{SO_{2}}$ in being restored to its initial baseline values (*p* > 0.05; paired *t*-test).

## Discussion

The present study used the fPAM technique to investigate the relative HbT and SO_2_ changes in a specific cross-section region of the rat sciatic nerve in response to temperature modulation by cooling and rewarming. Our main findings are as follows:

1) The functional HbT and SO_2_ changes in the rat sciatic nerve can be reliably investigated in a label-free manner using the described fPAM settings.

2) The functional HbT and SO_2_ changes were linearly correlated with temperature in both cooling and rewarming stages.

3) For the first time, the results suggest that relative HbT has a stronger correlation with localized nerve temperature changes in comparison to SO_2_ changes within the dynamic range of temperature modulation tested in this study.

### Temperature dependent vasculature hemodynamics in rat sciatic nerve

As shown in our experimented results, the presented fPAM setting is able to directly measure the relative HbT and SO_2_ changes at 32 × 61 μm resolution in an exposed nerve_._ This level of measurement cannot be directly and reliably detected by other existing imaging techniques [18,34]. fPAM can independently measure hemodynamic features according to the intrinsic optical absorption spectra [17,18], where *I*_*(570)*_ is used as a marker of relative HbT changes, and *I*_*F(560)*_ as a marker of relative SO_2_ changes [23].

Our findings show that both *R*_*HbT*_ and $R_{SO_{2}}$ in a selected ROI of the sciatic nerve decreased significantly during the cooling stage (Figure 3). Both changes in HbT and SO_2_ were significantly proportional to temperature changes (Figure 3) [35,36]. But it is also noted that $R_{SO_{2}}$ decreased to a lower relative value when compared to *R*_*HbT*_ and in response to cooling by about 10°C. These results correlate well with previous laser Doppler flowmetry studies [9,37] showing the clear relationship between the blood flow and tissue temperature [38]. It is intriguing to observe *R*_*HbT*_ being more strongly correlated with temperature modulation than $R_{SO_{2}}$ (Figure 3B and 3C). One plausible explanation is that there are two major effects resulting from nerve temperature changes: 1) vasoconstriction due to lowered temperatures and 2) metabolism down-regulation due to reduced temperature levels. A dominant effect of vasoconstriction over metabolism down-regulation results in more significant relative HbT change [39,40]. Nemoto et al. measured cortical evoked responses to somatosensory stimuli and showed that HbT-related signals are more highly correlated to the local region than oxygenation-related signals [41].

### Characteristics of the current functional PAM system compared to other imaging techniques

MUI is a useful method, which provides satisfactory indicators of morphological and blood flow changes [3], even in the nerve. But, MUI is limited by not being able to utilize intrinsic contrast for measurements of hemodynamic parameters. However, the relationship between morphological changes and their hemodynamics plays an important role for developing a better understanding of neurovascular functions. Thus, the development of the current fPAM system can be of significant benefit for the interpretation of MUI by registering intrinsic hemodynamic signals. Blood oxygen-level dependent (BOLD) fMRI is another method used to study hemodynamics, but it lacks selectivity between oxygen delivery and consumption, which may both occur during neuronal activation [42]. For instance, it is possible for a negative BOLD signal to occur under very high neuronal activity, where changes in oxygen consumption may far exceed oxygen supply [43]. Indeed, the interpretation of a given BOLD signal response may be more accurate [23,33] by combining it with fPAM to isolate the contribution of SO_2_ changes.

The 50 MHz ultrasound B-scan image provides a view of the complete cross-section of the sciatic nerve (Figure 2B), but only a part of it can be imaged by fPAM. This discrepancy is caused by the penetration depth of light in body tissue and blood [16,17]. Limited penetration depth of light in blood vessels restricts the maximum measurable axial diameter registered by the current fPAM system [33] as shown in (Figure 2C). To an extent, the dark-field illumination employed in fPAM system and the natural scattering of light in nerve tissue helps to increase the penetration of light in bio-tissue in this *in vivo* experiments [33]. The scope of this study includes only the temperature dependent hemodynamics visualized with fPAM for a ROI (Figure 3A). We would like to emphasize, that presently we are only considering relative and micro-resolution functional hemodynamic changes in the blood vessels of rat sciatic nerve, as opposed to absolute vasculature changes.

While there has been a lot interest in studying hemodynamics or neurovascular functions as well as the imaging of such coupling in relation to therapies such as hypothermia in the brain, very little has been done to study the neurovascular coupling in the peripheral nerves [44]. We aim to understand and develop the *in vivo* fPAM technique for the study of peripheral nervous system hemodynamics. The peripheral nerve system presents itself an ideal object of study since the nerves lie just millimeters below the skin, providing a simple and robust model to study hemodynamics in response to temperature changes.

Here, we report fundamental correlations between relative HbT and SO_2_ in response to temperature modulation via cooling and rewarming. Further investigation is needed to better understand the relationship between hemodynamics and neuronal activation under different modalities of temperature stimulation. In addition to temperature modulation of the nerve, it is worth mentioning that this technique may also be applicable to monitor the effects of various other *in vivo* stimulation protocols, such as electrical [23] and pharmacological. Most importantly we envisage this technique to be useful for determining blood flow dynamics in human neuropathies caused by vascular pathology such as diabetic neuropathy [11].

## Conclusions

In summary, our results show that the current fPAM technique is a good candidate for detecting the hemodynamic changes reflected in the relative HbT and SO_2_ fluctuations evoked by temperature changes in rat peripheral nerve. We found that changes in relative HbT are more linearly correlated to nerve temperature modulation than SO_2_. This finding suggests that the significant relative HbT changes are more co-localized with neuronal activation in the nerve. We also show that the regulation of sciatic nerve hemodynamics can be reliably studied by fPAM technique without the use of contrast agents. The technique we describe may help shed light on the neuroprotective effect of hypothermia on peripheral nerve by visualizing its intrinsic hemodynamics. Along this line, it may also add to our understanding of peripheral nerve hemodynamics and present important implications for the development of new therapeutic approaches.

## Abbreviations

fPAM: Functional photoacoustic microscopy; PA: Photoacoustic; HbT: Total hemoglobin concentration; SO2: Hemoglobin oxygen saturation; MUI: Medical ultrasound imaging; fMRI: Functional magnetic resonance imaging; HbO2: Oxy-hemoglobin; Hb: Deoxy-hemoglobin; SNR: Ratio of the signal peak value to the root-mean-square value of the noise; ANSI: American National Standards institute; IACUC: Institutional Animal Care and Use Committee; ROI: Region of interest; BOLD: Blood oxygen-level dependent.

## Competing interests

The authors declared that they have no competing interests.

## Authors’ contributions

Study concept and design (LDL, JO, KQS and EWS); drafting of the manuscript (LDL and JO); critical revision of the manuscript for important intellectual content (LDL, JO, AV, YHL, NT, KQS and EWS); obtained funding (NT, LDL); administrative, technical, and material support (LDL, JO, NT, KQS and EWS); study supervision (LDL, NT, AL, KQS and EWS). All authors read and approved the final manuscript.

## Supplementary Material

Additional file 1: Movie S1 This movie shows the response for both relative HbT changes as function of time under the thermoregulation protocol (i.e., cooling and rewarming stages). Click here for file
